# Supplementary material for: From sequence to enzyme mechanism using multi-label machine learning
Source: BMC Bioinformatics. 2014 May 19;15:150. doi: 10.1186/1471-2105-15-150 (PMC4229970; doi:10.1186/1471-2105-15-150)
Supplement: Additional file 2 — Java code of ml2db. Additional file ml2db_code.tar.gz contains the Java source code to run the multi-label machine learning experiments and save the results to database. The code’s Javadoc is included. [file 1471-2105-15-150-S2.zip › additional file 2/ml2db/ml2db/doc/main/ml2db/mulan/learn/traintest/TrainTestExperimenterSerialized.html]

TrainTestExperimenterSerialized


JavaScript is disabled on your browser.


- Overview
- Package
- Class
- Use
- Tree
- Deprecated
- Index
- Help

- Prev Class
- Next Class

- Frames
- No Frames

- All Classes

- Summary:
- Nested |
- Field |
- Constr |
- Method

- Detail:
- Field |
- Constr |
- Method


main.ml2db.mulan.learn.traintest

## Class TrainTestExperimenterSerialized

- java.lang.Object
- - main.ml2db.mulan.learn.crosseval.CrossExperimenter
  - - main.ml2db.mulan.learn.traintest.TrainTestExperimenter
    - - main.ml2db.mulan.learn.traintest.TrainTestExperimenterSerialized

- ---

    

  ```
  public class TrainTestExperimenterSerialized
  extends TrainTestExperimenter
  ```

  Executes a train/test evaluation using a previously trained model saved on
  file.

  Version:
  :   25 Oct 2010

  Author:
  :   Luna De Ferrari ldf(at)st-andrews.ac.uk

- - ### Constructor Summary

    Constructors

    Constructor and Description

    `TrainTestExperimenterSerialized(DbManagers dbMan,
    int trainArffRecordId,
    int testArffRecordId,
    MulanLearner learner,
    java.lang.String trainedModelFile,
    java.lang.String resultsDirectory)`
  - ### Method Summary

    Methods

    | Modifier and Type | Method and Description |
    | --- | --- |
    | `TrainTestEvaluator` | `getNewEvaluator()` |
    | `java.lang.String` | `getTrainedModelFile()` |
    | `static void` | `main(java.lang.String[] args)` |
    | `static void` | `runWithSerializedTrainedModel(DbManagers arffStoreAndResultsDbManagers, int trainArffRecordId, int testArffRecordId, MulanLearner learner, java.lang.String trainedModelFile, java.lang.String resultsFilesDirectory, boolean predict, boolean saveTrueNegatives)` |
    | `void` | `setTrainedModelFile(java.lang.String trainedModelFile)` |
    | `java.lang.String` | `toString()` |
    | `static java.lang.String` | `trainAndSerialize(int trainArffRecordId, MulanLearner learner, DbManagers dbManagers, java.lang.String directoryForSerializedModel)` |

    - ### Methods inherited from class main.ml2db.mulan.learn.traintest.TrainTestExperimenter

      `evaluateAndSave, getEvaluator, getInputManager, getOutputManager, run, setInputManager, setInputManager, setInputManager, setOutputManager`
    - ### Methods inherited from class main.ml2db.mulan.learn.crosseval.CrossExperimenter

      `evaluateAndSave, getLearner, getTestArffId, getTrainArffId, run, setTestArffId`
    - ### Methods inherited from class java.lang.Object

      `equals, getClass, hashCode, notify, notifyAll, wait, wait, wait`

- - ### Constructor Detail


    - #### TrainTestExperimenterSerialized

      ```
      public TrainTestExperimenterSerialized(DbManagers dbMan,
                                     int trainArffRecordId,
                                     int testArffRecordId,
                                     MulanLearner learner,
                                     java.lang.String trainedModelFile,
                                     java.lang.String resultsDirectory)
      ```
  - ### Method Detail


    - #### getNewEvaluator

      ```
      public TrainTestEvaluator getNewEvaluator()
      ```

      **Overrides:**
      :   `getNewEvaluator` in class `TrainTestExperimenter`


    - #### getTrainedModelFile

      ```
      public java.lang.String getTrainedModelFile()
      ```


    - #### setTrainedModelFile

      ```
      public void setTrainedModelFile(java.lang.String trainedModelFile)
      ```


    - #### toString

      ```
      public java.lang.String toString()
      ```

      **Overrides:**
      :   `toString` in class `java.lang.Object`


    - #### main

      ```
      public static void main(java.lang.String[] args)
      ```


    - #### runWithSerializedTrainedModel

      ```
      public static void runWithSerializedTrainedModel(DbManagers arffStoreAndResultsDbManagers,
                                       int trainArffRecordId,
                                       int testArffRecordId,
                                       MulanLearner learner,
                                       java.lang.String trainedModelFile,
                                       java.lang.String resultsFilesDirectory,
                                       boolean predict,
                                       boolean saveTrueNegatives)
      ```


    - #### trainAndSerialize

      ```
      public static java.lang.String trainAndSerialize(int trainArffRecordId,
                                       MulanLearner learner,
                                       DbManagers dbManagers,
                                       java.lang.String directoryForSerializedModel)
      ```


- Overview
- Package
- Class
- Use
- Tree
- Deprecated
- Index
- Help

- Prev Class
- Next Class

- Frames
- No Frames

- All Classes

- Summary:
- Nested |
- Field |
- Constr |
- Method

- Detail:
- Field |
- Constr |
- Method
